# The enantiospecific synthesis of (+)-monomorine I using a 5-*endo*-trig cyclisation strategy

**DOI:** 10.1186/1860-5397-3-39

**Published:** 2007-11-08

**Authors:** Malcolm B Berry, Donald Craig, Philip S Jones, Gareth J Rowlands

**Affiliations:** 1 Department of Chemistry, Imperial College London, South Kensington Campus, London, SW7 2AZ, UK; 2 Roche Discovery Welwyn, Broadwater Road, Welwyn Garden City, Hertfordshire AL7 3AY, UK; 3 Institute of Fundamental Sciences, Massey University, Private Bag 11 222, Palmerston North, New Zealand

## Abstract

We have developed a general strategy for the synthesis of 2,5-*syn* disubstituted pyrrolidines that is based on the multi-faceted reactivity of the sulfone moiety and a 5-*endo*-trig cyclisation. This methodology was applied to the synthesis of indolizidine alkaloid monomorine I. Two factors were key to the success of this endeavour; the first was the choice of nitrogen protecting group whilst the second was the conditions for the final stereoselective amination step. Employing a combination of different protecting groups and an intramolecular reductive amination reaction we were able to prepare (+)-monomorine I in just 11 steps from commercially available D-norleucine in a completely stereoselective manner.

## Background

**Scheme 1:**
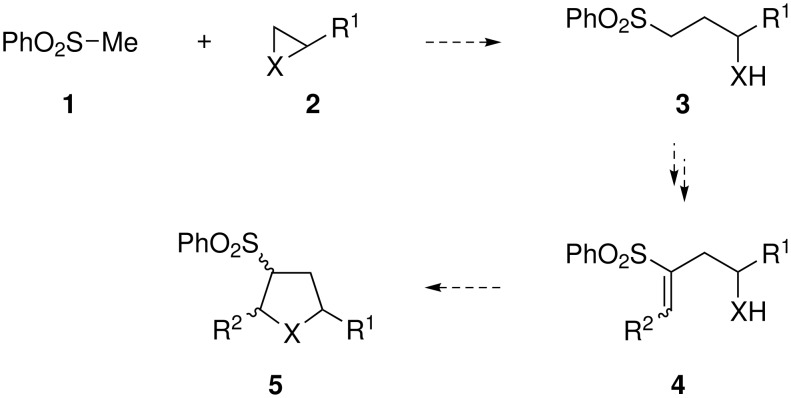
General strategy for the synthesis of heterocycles *via* 5-*endo*-trig cyclisation

The abundance in natural products and drug candidates of saturated five-membered heterocycles, such as tetrahydrofurans and pyrrolidines, makes these motifs attractive targets for synthesis. Over the last decade we have developed a powerful general strategy for the preparation of such compounds based upon the multi-faceted reactivity of the sulfone group and the formally disfavoured 5-*endo*-trig mode of cyclisation. [1–6] The methodology allows the conversion of epoxides (X = O) or aziridines (X = N-PG) (**2**) into the desired trisubstituted tetrahydrofurans or pyrrolidines (**5**) *via* a series of sulfone-mediated transformations (Scheme 1). Ring-opening **2** with the sulfone-stabilised anion of **1** forms the first C-C bond and furnishes **3**. Modification of the work of Julia [7–9] then utilises the sulfone to facilitate stereocontrolled alkenylation to give the cyclisation substrate **4**. Finally, 5-*endo*-trig cyclisation yields the desired heterocycles **5**. Overall, the sulfone moiety enables two C-C bond forming steps, allows stereocontrol of the alkene and activates the alkene to cyclisation. Furthermore, the sulfone can be used to elaborate the basic framework post-cyclisation.

In this publication we outline the application of this methodology to the synthesis of the indolizidine, (+)-monomorine I. [10–13] We have briefly described this work in a previous communication. [4]

## Results and Discussion

**Scheme 2:**
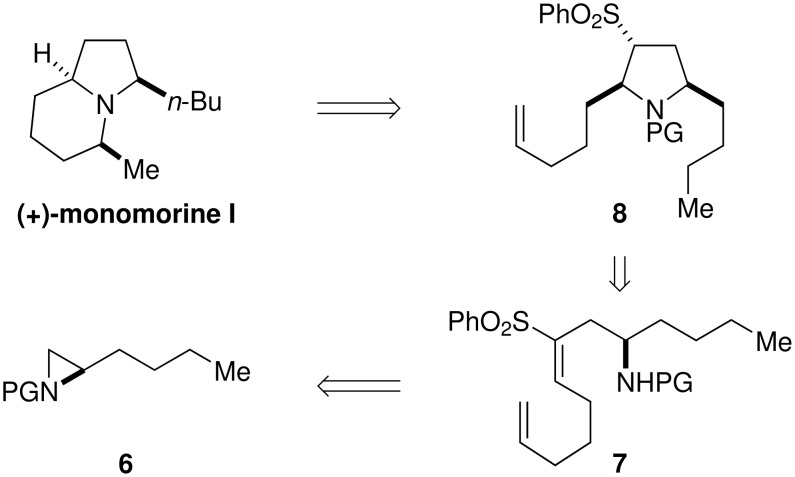
Retrosynthesis of (+)-monomorine I

The pyrrolidine ring is an important structural motif that occurs in a range of pheromones, venoms and drug candidates. [14] In order to demonstrate the synthetic utility of the sulfone-mediated 5-*endo*-trig methodology. [3] we embarked on the total synthesis of the indolizidine alkaloid monomorine I, the trail pheromone of the Pharaoh worker ant *Monomorium pharaonis*. [10] Our initial synthetic plan is outlined in Scheme 2; aziridine **6**, prepared from D-norleucine by standard transformations, would be converted into the 2,5-*syn* disubstituted pyrrolidine core **8** *via* alkene **7**. With all the required carbon atoms in place, the final steps would involve deprotection, intramolecular hydroamination of the alkene and desulfonylation.

Initial studies directed towards this goal exploited the tosyl moiety as the nitrogen-protecting group (PG) and resulted in a succinct synthesis of alkenes of the type **4** (X = NTs; Scheme 1). [15] Disappointingly, all attempts to ring-close the sulfonamides proved fruitless, and it was found that desulfonylation was necessary before cyclisation could be achieved. Whilst the tosyl-based methodology permitted the synthesis of a range of simple, non-functionalised pyrrolidines **5** (X = NH), the harsh nature of the deprotection reaction, treatment with hydrobromic acid and phenol in acetic acid at reflux, led to the destruction of the the terminal alkene functionality of **7** (PG = Ts; Scheme 2) required for our synthesis of (+)-monomorine I. As a result of this set-back, a second nitrogen protecting group was assessed. The diphenylphosphinyl group (PG = P(O)Ph_2_ = Dpp) overcame many of the problems encountered with the tosyl group; protected alkenes **4** (X = NDpp) underwent smooth 5-*endo*-trig cyclisation to furnish *N*-(diphenylphosphinyl)pyrrolidines **5** (X = Dpp) in good yields. [3,16] Furthermore, dephosphinylation was readily achieved under either Lewis acidic or Brønsted acid conditions compatible with a range of functional groups. This second-generation methodology was limited by the finding that acylation of **3** (X = NDpp) could only be achieved with non-enolisable acid chlorides, rendering it unsuitable for the synthesis of (+)-monomorine I. Ultimately, no single protecting group was found to be suitable and it was necessary to exploit a combination of protecting groups. The full evolution of the 5-*endo*-trig cyclisation-based pyrrolidine methodology will be described in a future publication.

**Scheme 3:**
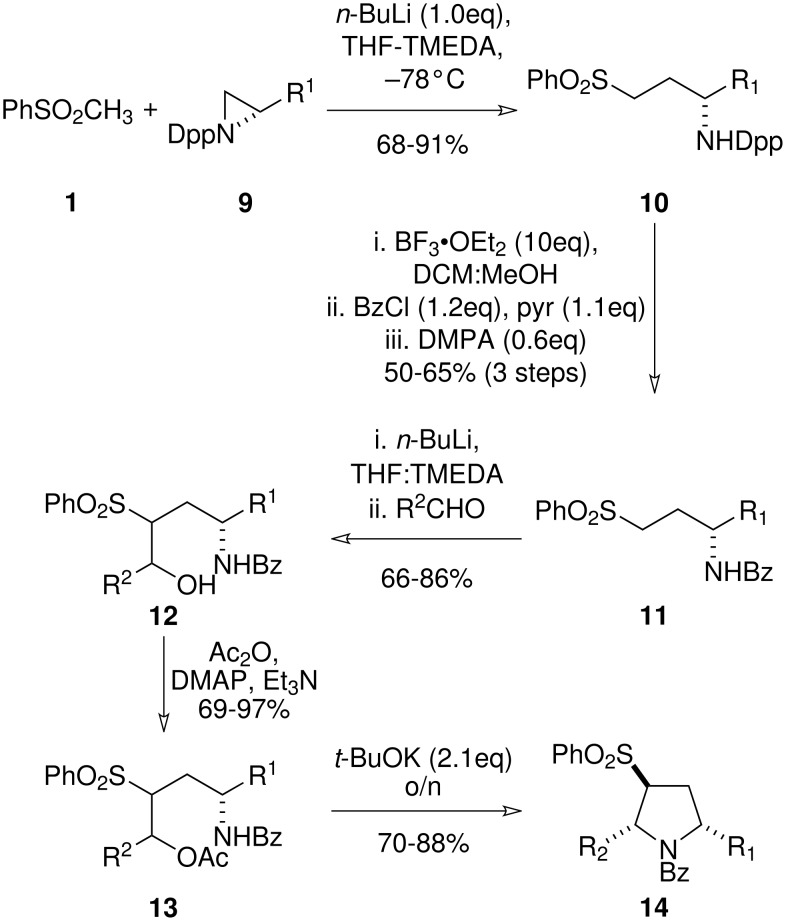
The sulfone-mediated synthesis of 2,5-*syn* disubstituted pyrrolidines *via* a 5-*endo*-trig cyclisation

Key to the successful synthesis of (+)-monomorine I was the use of the *N*-(benzoyl)aminosulfone **11** (Scheme 3). Benzamide **11** could be prepared from *N*-(diphenylphosphinyl)aziridine **9** by ring-opening with **1** followed by protecting group interchange. Although this strategy was not as elegant as utilising an *N*-benzoylaziridine directly, we deemed it prudent not to subject such a species to nucleophilic attack due to reported issues with chemoselectivity. [17] Careful optimisation obviated the need for chromatography following the protecting group exchange, and the benzamides **11** could be isolated in high purity and good yield. Hydroxyalkylation with a range of aldehydes proceeded without issue to give the β-hydroxysulfones **12** in excellent yields. The β-hydroxysulfones were then acylated under standard conditions to give **13**. Treatment of the β-acetoxysulfones **13** with two equivalents of base gave the pyrrolidines **14** directly as the product of a one-pot elimination-cyclisation cascade. The pyrrolidines were formed with complete diastereoselectivity for the 2,5-*syn* diastereoisomers. Although this stereochemical relationship could not be discerned from the ^1^H NMR spectra of **14** due to peak broadening caused by amide rotamers, a combination of further elaboration and X-ray crystallographic analysis confirmed the assignment.

**Scheme 4:**
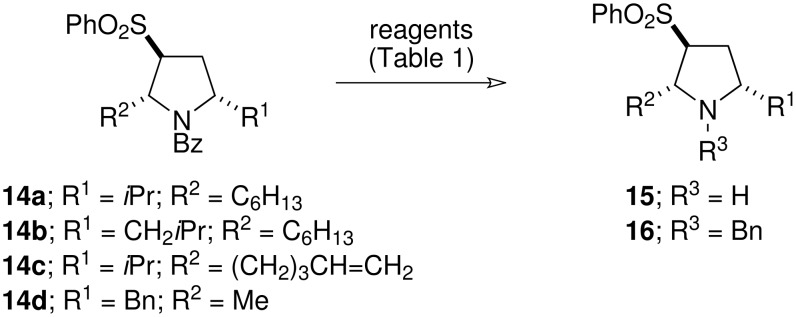
The deprotection of *N*-benzoylpyrrolidines

Deprotection of simple benzoyl-protected pyrrolidines **14a** and **14b** could be achieved by acid hydrolysis (Scheme 4 and Table 1). However, as with the tosyl-based methodology, such reaction conditions were incompatible with the terminal alkene-substituted pyrrolidine **14c**. Therefore alternative deprotection conditions were investigated. Attempted base-mediated hydrolysis led to formation of the *N*-benzoylaminosulfone **11**, presumably by a sequence involving ring-opening by elimination, hydration of the electron-deficient alkenyl sulfone double bond and retro-aldol-like fragmentation. Reductive deprotection proved to be a more fruitful avenue of study. After considerable optimisation it was found that treatment of the *N*-benzoylpyrrolidines with Super-Hydride^®^[18] gave the free amines **15**, whilst the use of DIBAL in THF furnished the benzyl-protected pyrrolidines **16** in good yield (Scheme 4 and Table 1).

**Table 1:** Deprotection of *N*-benzoylpyrrolidines

**Pyrrolidine**	**R** ^1^	**R** ^2^	**Reagent**	**R** ^3^	**Product**	**Yield (%)**

**14a**	*i*Pr	C_6_H_13_	HCl	H	**15a**	69
**14b**	CH_2_*i*Pr	C_6_H_13_	HCl	H	**15b**	60
**14a**	*i*Pr	C_6_H_13_	Super-Hydride^®^	H	**15a**	69
**14c**	*i*Pr	(CH_2_)_3_CH = CH_2_	Super-Hydride^®^	H	**15c**	57
**14c**	*i*Pr	(CH_2_)_3_CH = CH_2_	DIBAL	Bn	**16c**	70
**14d**	Bn	Me	DIBAL	Bn	**16d**	67

**Scheme 5:**
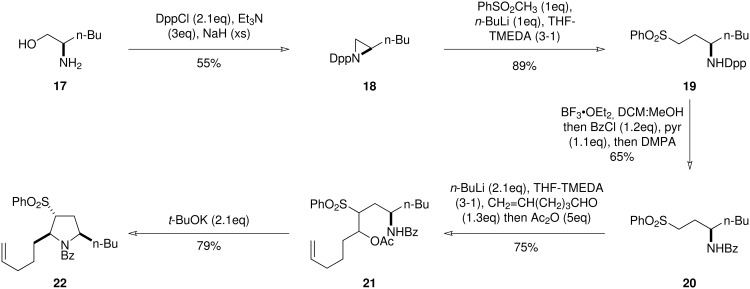
Synthesis of *N*-benzoyl protected pyrrolidine required for the preparation of (+)-monomorine I

With the chemistry now in place to undertake the synthesis of (+)-monomorine I, the initial target, pyrrolidine **22**, was prepared. Commercially available D-norleucine was reduced to the amino alcohol **17**. [19] This was then converted into the benzoyl-protected aminosulfone **20** *via* the diphenylphosphinylaziridine **18,** which was ring-opened to give **19**, followed by protecting group exchange (Scheme 5). Formation of the dianion of **20** by exposure to two equivalents of *n*-butyllithium, followed by reaction with hex-5-enal and *in situ* trapping of the intermediate alkoxides gave the ester **21** as predominantly one diastereoisomer. Finally, one-pot elimination-cyclisation, promoted by two equivalents of potassium *tert*-butoxide, furnished the 2,5-*syn*-pyrrolidine **22** as a single diastereoisomer. Concurrently with the synthesis of **22**, the *iso*propyl model system, **14c**, was prepared using analogous chemistry.

**Scheme 6:**
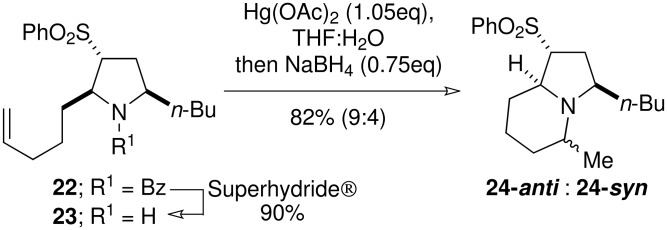
Mercury-mediated hydroamination

**Scheme 7:**
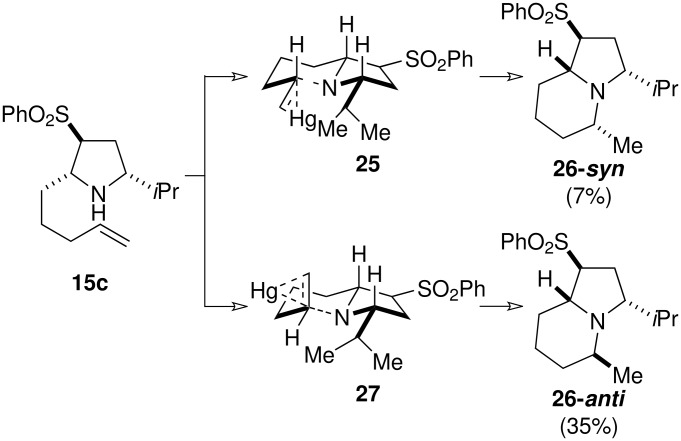
Proposed transition state for mercury-mediate hydroamination

Deprotection of **22** and **14c** was readily achieved with Super-Hydride^®^ to give the free amines **23** and **15c**, which were subjected to mercury-mediated hydroamination (Scheme 6 and Scheme 7). [20] Cyclisation of **23** proceeded in good yield to give a 9:4 mixture of two indolizidines, epimeric at the C-5 methyl group **24-*anti*** and **24-*syn*** (Scheme 6). Cyclisation of the *iso*propyl analogue **15** proceeded with improved stereoselectivity to give a 5:1 mixture of epimeric indolizidines **26-*anti*** and **26-*syn*** (Scheme 7). Presumably, the increased steric bulk of the *iso*propyl group is responsible for the higher *anti*-selectivity. Assignment of the relative stereochemistry of the epimeric pairs proved problematic due to difficulties encountered during separation, and the presence of overlapping signals in the ^1^H NMR spectrum. Finally, a combination of X-ray diffraction analysis and comparison of the ^1^H NMR showed that the major diastereoisomer in each case was the undesired C-5 epimer, with the methyl group residing in the axial position. Naturally, we had assumed that the diastereoisomer in which all the substituents adopted a pseudo-equatorial orientation would have been formed preferentially. Yet inspection of the possible transition states for the cyclisation **25** *vs*. **27** reveals that the axial methyl may be favoured so as to minimize the strain associated with the eclipse of the C-3 and C-5 substituents (Scheme 7). Branching of the *iso*propyl substituent would cause greater interaction than the butyl group, and therefore would lead to an increase in selectivity.

**Scheme 8:**
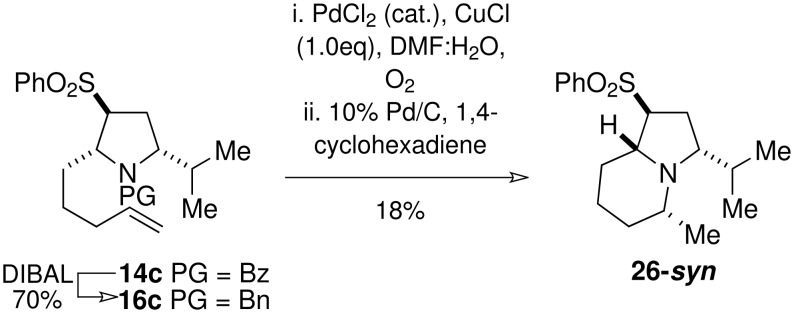
Model study for the reductive amination-based cyclisation

**Scheme 9:**
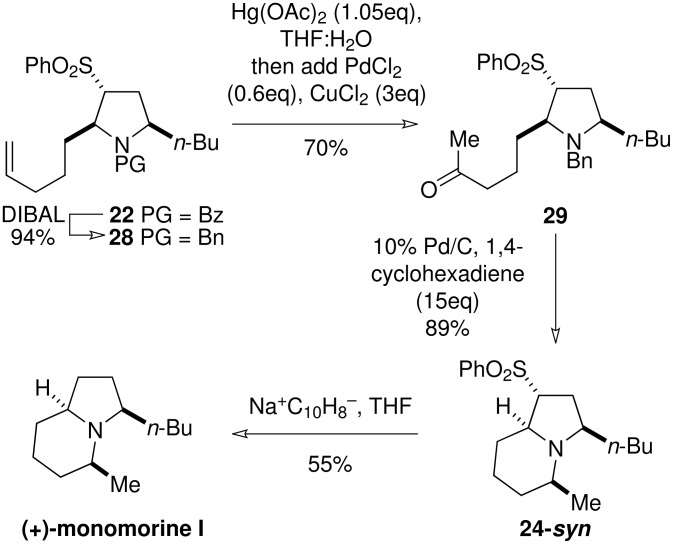
The synthesis of (+)-monomorine I

The findings described above dictated that an alternative cyclisation strategy be investigated. It was anticipated that intramolecular reductive amination of a pendant methyl ketone would furnish the correct diastereoisomer, because the hydride source would be expected to approach the iminium ion from the less sterically demanding face, with the C-9 stereocentre being the controlling factor. [21] Both the benzoyl protecting group and the free amine were deemed incompatible with such a strategy. Therefore, **22** and **14c** were converted into the benzyl-protected pyrrolidines **28** and **16c** respectively by partial reduction with DIBAL-H (Scheme 8 and Scheme 9). Wacker oxidation[22] of the *iso*propyl model compound **16c** gave the desired methyl ketone, which was subjected to transfer hydrogenation. [23] The latter reaction precipitated a reaction cascade commencing with deprotection of the *N*-benzylpyrrolidine followed by intramolecular reductive amination to give the desired indolizidine **26-*syn*** as a single diastereoisomer in 18% yield for the two steps. Whilst the yield of this unoptimised reaction was not satisfactory, we were pleased to observe that only the desired diastereoisomer was formed.

Oxidation of the terminal alkene of **28** under Wacker conditions proved highly capricious and was ultimately abandoned in favour of a more reliable oxymercuration protocol. [24] Under these conditions the methyl ketone **29** was isolated in 70% yield (Scheme 9). Catalytic transfer hydrogenation led to sequential debenzylation and intramolecular reductive amination to furnish **24-*syn*** as a single diastereoisomer in excellent yield. Desulfonylation was achieved by brief exposure of **24-*syn*** to sodium naphthalenide in THF to furnish (+)-monomorine I, which showed ^1^H and ^13^C NMR, IR, mass spectral and optical rotation characteristics in agreement with published values. [25] Short reaction times were found to be crucial to the success of this reaction.

In summary, we have developed a highly stereoselective 5-*endo*-trig cyclisation reaction that facilitates the preparation of 2,5-*syn* disubstituted pyrrolidines. We have used this transformation as the key step in the synthesis of the indolizidine alkaloid, (+)-monomorine I. The synthesis was achieved in nine steps from the readily available aziridine **18**, and compares favourably with other total syntheses in the literature.

See Supporting Information File 1 for full experimental data.

## Supporting Information

File 1 The enantiospecific synthesis of (+)-monomorine I using a 5-*endo*-trig cyclisation strategy: full experimental data. Full preparative details of all compounds prepared are reported, together with their spectroscopic data.
