# Supplementary material for: The enantiospecific synthesis of (+)-monomorine I using a 5-endo-trig cyclisation strategy
Source: Beilstein J Org Chem. 2007 Nov 8;3:39. doi: 10.1186/1860-5397-3-39 (PMC2151076; doi:10.1186/1860-5397-3-39)
Supplement: File 1 — The enantiospecific synthesis of (+)-monomorine I using a 5-endo-trig cyclisation strategy: full experimental data. Full preparative details of all compounds prepared are reported, together with their spectroscopic data. [file Beilstein_J_Org_Chem-03-39-s001.doc]

**The enantiospecific synthesis of (+)-monomorine I using a 5-*endo*-trig cyclisation strategy**

Malcolm B. Berry,^1,2^ Donald Craig,*^1^ Philip S. Jones^3,4^ and Gareth J. Rowlands^1,5^

^1^ Department of Chemistry, Imperial College London, South Kensington Campus, London, SW7 2AZ, U.K. Email: [d.craig@imperial.ac.uk](mailto:d.craig@imperial.ac.uk).

^2^ Present email address: [malcolm.b.berry@gsk.com](mailto:malcolm.b.berry@gsk.com).

^3^ Roche Discovery Welwyn, Broadwater Road, Welwyn Garden City, Hertfordshire AL7 3AY, U.K.

^4^ Present email address: [p.jones@organon.co.uk](mailto:p.jones@organon.co.uk).

^5^ Present address: Institute of Fundamental Sciences, Massey University, Private Bag 11 222, Palmerston North, New Zealand. Email: [g.j.rowlands@massey.ac.nz](mailto:g.j.rowlands@massey.ac.nz).

**Additional file 1**

**Full experimental data**

^1^H NMR spectra were recorded in CDCl_3_ on either Bruker AM-500, Bruker DRX-300, Bruker DRX-400, Bruker AM-400, Jeol GX-270Q or Bruker AC-250 spectrometers, using residual isotopic solvent (CHCl_3_, δ_H_ = 7.26 ppm or δ_H_ = 7.28 ppm on Bruker DRX-300) as internal reference. First-order multiplets were assigned according to Hoye.[1] Infra-red spectra were recorded on a Perkin-Elmer 881 or Mattson 5000 FTIR spectrophotometer. Mass spectra were obtained using Jeol DX-303, VG-7070B, VG 12-253 and VG ZAB-E instruments. Elemental combustion analyses were performed in the Imperial College Chemistry Department microanalytical laboratory. Melting points are measured on a Reichert hot stage apparatus and are uncorrected. Optical rotations were measured using a Perkin-Elmer 241 polarimeter. Air- and moisture-sensitive reagents were transferred *via* syringe or cannula, and reactions involving these materials were carried out in oven-dried flasks under a positive pressure of nitrogen. Chromatography refers to flash column chromatography on Merck Kieselgel 60 (230-400 mesh) unless otherwise stated. Tlc refers to analytical thin-layer chromatography performed using pre-coated glass-backed plates (Merck Kieselgel 60 F_254_) and visualised with ultraviolet light, iodine, acidic ammonium molybdate (IV), acidic ethanolic vanillin, aqueous potassium manganate(VII) or acidic anisaldehyde as appropriate. Petrol refers to redistilled 40°-60° petroleum ether, and ether to diethyl ether. Ether and THF were distilled from sodium-benzophenone ketyl, CH_2_Cl_2_ from phosphorus pentoxide and toluene from sodium. Where appropriate, reagents were purified before use according to standard procedures.[2]

**(2*R*)-1-Diphenylphosphinyl-2-butylaziridine (18).**

To a solution of (*R*)-2-aminohexan-1-ol (11.51 g, 98.30 mmol, 1.0 eq.) in THF (330 mL) at 0 °C under N_2_ was added Et_3_N (41.3 mL, 294.9 mmol, 3.0 eq.) and diphenylphosphinic chloride (39.4 mL, 206.4 mmol, 2.1 eq.). After addition of 1 eq. of diphenylphosphinic chloride a white precipitate was formed. The suspension was stirred for 20 h at rt. Excess NaH (> 10 equiv) was added in two portions and the grey suspension stirred for two days. The suspension was cooled to 0 °C and ice was added cautiously over 30 min. The reaction mixture was filtered through MgSO_4_ and the residue washed with Et_2_O (2 x 150 mL). The solvent was concentrated under reduced pressure and the resultant brown oil purified by chromatography (60% EtOAc / petrol) to give **18** as a colourless oil (16.16 g, 55%); [α]_D_^27^ +13.5 (*c* 1.28, CH_2_Cl_2_); ν_max_ (film) 2956, 2930, 2871, 2872, 1438, 1191, 1126, 1110, 728 and 687 cm^–1^; δ_H_ (300 MHz) 7.92-7.85 (4H, m, Ar-H), 7.44-7.32 (6H, m, Ar-H), 2.65 (1H, dddd, *J =* 15.5, 12.0, 6.0, 5.5 Hz, H-2), 2.56 (1H, ddd, *J =* 17.5, 6.0, 1.0 Hz, H-3), 1.94 (1H, ddd, *J =* 11.5, 3.5, 1.0 Hz, H-3), 1.55-1.48 (2H, m, 2 x H-1’), 1.30-1.14 (4H, m, 2 x H-2’ and 2 x H-3’), 0.79 (3H, t, *J =* 7.0 Hz, H-4’); δ_C_ (75 MHz) 133.6, 133.5, 132.6, 132.5, 132.0, 131.7, 131.7, 131.6, 131.5, 128.5, 128.4, 128.3, 128.3, 128.2, 35.5 (d), 32.2 (d), 29.6 (d), 28.9, 22.2, 13.8; *m/z* (CI) 599 [2M+H]^+^, 300 [M+H]^+^, 275, 247, 218, 203, 98 (Found: [M+H]^+^, 300.1494. C_18_H_22_NOP requires [M+H]^+^, 300.151728); (Found: C, 72.33; H, 7.07; N, 4.95. C_18_H_22_NOP requires C, 72.24; H, 7.36; N, 4.68%).

**(3*R*)-3-(Diphenylphosphinyl)amino-1-(phenylsulfonyl)heptane (19).**

To an oven dried flask at *ca.* 150 °C containing a magnetic stirrer-bar was added (phenylsulfonyl)methane (3.19 g, 20.4 mmol, 1.0 eq.). The flask containing the melt was immediately fitted with a rubber septum and allowed to cool under a stream of dry nitrogen. 75% THF-TMEDA (51.1 mL) was added and the solution cooled to –78 °C. To the resulting white suspension *n*-BuLi (2.5 M in hexanes; 9.81 mL, 24.53 mmol, 1.2 eq.) was added dropwise. The solution cleared to a golden yellow colour and was stirred at –78 °C for 10 min, whereupon a solution of (2*S*)-1-diphenylphosphinyl-2-(1-methylethyl)aziridine **18** (5.50 g, 18.4 mmol, 0.9 eq.) in THF (18.40 mL) was added. The solution darkened considerably. After a further 15 min at –78 °C the mixture was warmed to rt. overnight. The reaction mixture was partitioned between saturated aqueous NH_4_Cl (50 mL) and CH_2_Cl_2_ (100 mL). The aqueous layer was extracted with EtOAc (3 x 50 mL). The combined organic layers were dried (MgSO_4_) and concentrated under reduced pressure to give a viscous yellow oil. The oil was adsorbed on SiO_2_ and purified by chromatography (2% MeOH / CH_2_Cl_2_) to give **19** as a white solid (7.44 g, 89%); mp 134-136°C; [α]_D_^27^ +12.2 (*c* 0.58, CHCl_3_); ν_max_ (Nujol) 3445, 3222, 1636, 1425, 1275, 1180, 1090, 1060, 730, 700 and 680 cm^–1^; δ_H_ (500 MHz) 7.92-7.39 (15H, m, Ar-H), 3.50 (1H, ddd, *J =* 15.0, 10.0, 5.0 Hz, H-1), 3.18 (1H, ddd, *J =* 17.5, 15.0, 5.0 Hz, H-1), 3.16-3.05 (1H, m, H-3), 2.77 (1H, dd, *J =* 10.0, 7.5 Hz, N-H), 2.05-1.96 (1H, m, H-2), 1.84-1.74 (1H, m, H-2), 1.59-1.42 (2H, m, 2 x H-4), 1.28-1.13 (4H, m, 2 x H-5 and 2 x H-6), 0.83 (3H, t, *J =* 7 Hz, Me); δ_C_ (126 MHz) 139.3, 133.5, 132.2, 132.1, 131.8, 131.7, 131.6, 129.2, 128.5, 128.5, 128.4, 128.3, 127.4, 53.0, 50.3, 37.3, 29.1, 27.5, 22.3, 13.2; *m/z* (electrospray) 456 [M+H]^+^, 440, 391, 317, 292, 186, 157; *m/z* (FAB^+^) 456 [M+H]^+^, 256, 219, 201, 143, 97, 86, 55 (Found: [M+H]^+^, 456.1779. C_25_H_30_NO_3_PS requires [M+H]^+^, 456.1763); (Found: C, 65.68; H, 6.43; N, 2.93. C_25_H_30_NO_3_PS requires C, 65.93; H, 6.59; N, 3.07%).

**(3*R*)-3-Benzoylamino-1-(phenylsulfonyl)heptane (20).**

To a solution of (3*R*)-3-(diphenylphosphinyl)amino-1-(phenylsulfonyl)heptane **19** (7.52 g, 23.95 mmol, 1.0 eq.) in 50% CH_2_Cl_2_ / MeOH (246 mL) at 0 °C under a nitrogen atmosphere was added BF_3_·OEt_2_ (29.86 mL, 239.50 mmol, 10.0 eq.). The solution was stirred at rt overnight then 2 M HCl (80 mL) was added. The layers were separated and the aqueous layer washed with EtOAc (2 x 50 mL). The combined organic layers were washed with 2 M HCl (2 x 20 mL) then discarded and the combined aqueous layers basified to pH 11 with 2 M NaOH. The basic aqueous layers were extracted with EtOAc (3 x 80 mL). The combined organic layers were dried (MgSO_4_) and concentrated under reduced pressure. The crude amine was taken on without further purification and dissolved in CH_2_Cl_2_ (160 mL). To the solution at 0˚C under an atmosphere of nitrogen was added pyridine (1.82 mL, 22.27 mmol, 1.1 eq.) and benzoyl chloride (2.81 mL, 24.29 mmol, 1.2 eq.). The solution was stirred at rt overnight before being cooled to 0˚C and 3-dimethylaminopropylamine (1.53 mL, 12.12 mmol, 0.6 eq.) added. The resultant white suspension was stirred at rt for 30 min after which it was partitioned between H_2_O (50 mL) and EtOAc (80 mL). The aqueous phase was extracted with EtOAc (3 x 50 mL). The combined organic layers washed with NH_4_Cl (50 mL) and brine (50 mL) three times then dried (MgSO_4_) and concentrated under reduced pressure to give, without further purification, **20** as a white solid (5.55 g, 65% two steps); mp 146-148˚C; [α]_D_ –28.40 (*c* 0.25, CHCl_3_); ν_max_ (CH_2_Cl_2_ film) 3328, 2960, 2923, 1785, 1632, 1602, 1579, 1531, 1491, 1445, 1305, 1212, 1145, 1084, 742, 692 and 668 cm^–1^; δ_H_ (300 MHz) 7.89 (2H, d, *J =* 7.0 Hz, *ortho*-Ar), 7.77 (2H, d, *J =* 7.0 Hz, *ortho*-Ar), 7.67 (1H, t, *J =* 7.5 Hz, *para*-Ar), 7.59-7.51 (3H, m, Ar-H), 7.47 (2H, t, *J =* 7.5 Hz, *meta*-Ar), 6.06 (1H, d, *J =* 9.0 Hz, N-H), 4.27-4.14 (1H, m, H-3), 3.34-3.08 (2H, m, 2 x H-1), 2.20-2.07 (1H, m, H-2), 2.06-1.90 (1H, m, H-2), 1.80-1.55 (3H, m, 3 x H of CH_2_), 1.36-1.28 (3H, m, 3 x H of CH_2_), 0.92-0.87 (3H, m, Me); δ_C_ (75 MHz) 167.37, 134.55, 133.81, 132.25, 131.77, 131.70, 131.63, 130.60, 129.38, 128.90, 128.68, 128.51, 127.97, 126.90, 53.58, 48.59, 35.27, 28.16, 28.11, 22.48, 13.95; *m/z* (CI) 377 [M+NH_4_]^+^, 360 [M+H]^+^, 220, 190, 160, 139, 122, 105, 86, 58 (Found: [M+H]^+^, 360.160966. C_20_H_25_NO_3_S requires [M+H]^+^, 360.163341).

**(9*R*)-6-Acetoxy-9-benzoylamino-7-(phenylsulfonyl)-1-tridecene (21).**

To a solution of (3*R*)-3-benzoylamino-1-(phenylsulfonyl)heptane **20** (0.18 g, 0.49 mmol, 1.0 eq.) in 75% THF–TMEDA at –78 °C under a nitrogen atmosphere was added *n*-BuLi (2.5 M in hexanes; 0.42 mL, 1.04 mmol, 2.1 eq.) to give an orange solution. The solution was stirred at –78˚C for 10 min whereupon 5-hexenal (1.0 M in THF; 0.59 mL, 0.59 mmol, 1.2 eq.) was added dropwise. The almost clear solution was stirred at –78 °C for 1 h after which Ac_2_O (0.23 mL, 2.47 mmol, 5.0 eq.) was added and the solution warmed to rt overnight. The resultant red solution was poured in to a vigorously stirred saturated NaHCO_3_ solution and NaHCO_3_ added until effervescence had ceased. The aqueous phase was extracted with Et_2_O (3 x 10 mL). The combined organics were dried (MgSO_4_) and evaporated under reduced pressure to give a yellow oil. Purification by chromatography (33% EtOAc / petrol) gave predominantly one diastereoisomer of **21** as a clear oil (0.18 g, 75%); ν_max_ (film) 3373, 3295, 3066, 2956, 2930, 2860, 1741, 1661, 1642, 1579, 1531, 1489, 1447, 1372, 1306, 1231, 1148, 1038, 1027, 999, 911, 720 and 691 cm^–1^; δ_H_ (300 MHz) 7.92 (2H, dd, *J =* 8.0, 1.5 Hz, *ortho*-Ar minor), 7.78 (2H, d, *J =* 7.0 Hz, *ortho*-Ar major), 7.76 (2H, d, *J =* 6.5 Hz, *ortho*-Ar major), 7.65 (2H, dd, *J =* 7.0, 1.5 Hz, *ortho*-Ar minor), 7.62 (2H, t, *J =* 7.5 Hz, 2 x *para*-Ar major), 7.59-7.33 (6H, m, Ar-H minor), 7.48 (2H, t, *J =* 8.0 Hz, *meta*-Ar major), 7.43 (2H, t, *J =* 7.5 Hz, *meta*-Ar major), 6.83 (1H, d, *J =* 9.0 Hz, N-H minor), 6.21 (1H, d, *J =* 9.5 Hz, N-H major), 5.78 (1H, dddd (ddt), *J =* 17.0, 10.5, 6.5, 6.5 Hz, H-2 major), 5.62 (1H, dddd (ddt), *J =* 17.0, 10.0, 6.5, 6.5 Hz, H-2 minor), 5.17 (1H, td, *J =* 7.0, 1.2 Hz, H-6 minor), 5.10 (1H, dt, *J =* 6.5, 3.0 Hz, H-6 major), 5.02 (1H, dd, *J =* 15.5, 2.0 Hz, H-1 major), 4.99-4.94 (1H, m, H-1 major), 4.88-4.80 (2H, m, 2 x H-1 minor), 4.38-4.24 (1H, m, H-9 major), 4.27-4.15 (1H, m, H-9 minor), 3.48 (1H, ddd (dt), *J =* 8.0, 3.0, 3.0 Hz, H-7 major), 3.15-3.13 (1H, m, H-7 minor), 2.28 (1H, dd, *J =* 10.0, 4.0 Hz, H-8 minor), 2.25-1.97 (4H, m, 2 x H-8 major, H-5 major & H-10 major), 2.07-1.81 (3H, m, H-8 minor & 2 x H of CH_2_ minor), 1.97 (3H, s, MeCO minor), 1.91 (3H, s, MeCO), 1.88-1.69 (2H, m, H-5 major & H-10 major), 1.74-1.55 (4H, m, 4 x H of CH_2_ minor), 1.58-1.49 (2H, m, 2 x H-4 major), 1.50-1.28 (4H, m, 4 x H of CH_2_ minor), 1.47-1.24 (6H, m, 3 x CH_2_ major), 1.26-1.20 (2H, m, 2 x H of CH_2_ minor), 0.94 (3H, t, *J =* 7.0 Hz, Me minor), 0.90 (3H, t, *J =* 7.0 Hz, Me major); δ_C_ (75 MHz) major 170.29, 167.39, 137.96, 133.82, 131.51, 129.17, 128.53, 128.45, 128.15, 127.12, 126.92, 115.01, 71.17, 63.38, 48.35, 35.94, 33.08, 29.70, 29.15, 27.94, 25.06, 22.46, 20.70, 13.90; minor 170.03, 167.41, 137.61, 137.14, 133.91, 131.62, 129.23, 128.94, 128.64, 127.02, 115.24, 68.92, 63.74, 49.95, 35.94, 32.74, 32.18, 27.94, 27.00, 24.28, 22.61, 20.88; *m/z* (CI) 517 [M+NH_4_]^+^, 500 [M+H]^+^, 440, 360, 300, 220, 204, 190, 160, 139, 122, 105, 86, 58 (Found: [M+H]^+^, 500.246223. C_28_H_37_NO_5_S requires [M+H]^+^, 500.247071).

**(2*S*,3*R*,5*R*)-1-Benzoyl-5-butyl-2-(4'-pentenyl)-3-(phenylsulfonyl)pyrrolidine (22).**

To a solution of (9*R*)-6-acetoxy-9-benzoylamino-7-(phenylsulfonyl)-1-tridecene **21** (0.56 g, 1.11 mmol, 1.0 eq.) in THF (33.7 mL) under a nitrogen atmosphere was added *t*-BuOH (1.05 mL, 11.13 mmol, 10.0 eq.) and *t*-BuOK (1 M in THF; 2.28 mL, 2.28 mmol, 2.05 eq.). The solution was stirred at rt overnight and gradually became a dark red colour. Saturated NH_4_Cl solution (50 mL) was added and the two phases separated. The aqueous layer was extracted with Et_2_O (3 x 20 mL) and the combined organics dried (MgSO_4_) and concentrated under reduced pressure to give a yellow oil. Purification of the oil by chromatography (33% EtOAc / petrol) gave **22** as a crystalline solid (0.39 g, 79%); mp 79-82˚C; [α]_D_^32^ +20.10 (*c* 1.03, CH_2_Cl_2_); ν_max_ (CH_2_Cl_2_ film) 3071, 3052, 2955, 2931, 2859, 2931, 2859, 1629, 1604, 1446, 1404, 1306, 1291, 1146, 1084, 733, 702 and 690 cm^–1^; δ_H_ (300 MHz) all broad due to rotamers 7.98-7.16 (10H, brm, Ar-H), 5.67 (1H, brs, H-4'), 4.91 (2H, brd, *J =* 10.0 Hz, H-5'), 4.67 (1H, brs, H-2), 4.07 (1H, brs, H-5), 3.44 (1H, brs, H-3), 2.83-2.80 (1H, m, H-4), 2.11-2.05 (1H, m, H-4), 1.98-0.72 (15H, brm, 6 x CH_2_ & Me); δ_C_ (75 MHz) broad 137.15, 134.07, 129.46, 129.37, 128.85, 128.34, 127.08, 126.41, 114.98, 66.44, 36.75, 32.82, 30.31, 27.79, 25.22, 22.46, 21.87, 13.77; *m/z* (CI) 440 [M+H]^+^, 300, 175, 160, 139, 105 (Found: [M+H]^+^, 440.227836. C_26_H_33_NO_3_S requires [M+H]^+^, 440.225941); (Found C, 70.78; H, 7.44; N, 3.16. C_26_H_33_NO_3_S requires C, 71.07; H, 7.52; N, 3.19%).

**(2*S*,3*R*,5*R*)-5-Butyl-2-(4'-pentenyl)-3-(phenylsulfonyl)pyrrolidine (23).**

To a solution of LiEt_3_BH (1M in THF; 0.87 mL, 0.87 mmol, 2.2 eq.) at rt under a nitrogen atmosphere was added a solution of (2*S*,3*R*,5*R*)-1-benzoyl-5-butyl-2-(4'-pentenyl)-3-(phenylsulfonyl)pyrrolidine **22** (0.17 g, 0.39 mmol, 1.0 eq.) in THF (0.8 mL) dropwise. The solution was stirred at rt for 8 h whereupon MeOH was carefully added until effervescence had ceased. The solution was poured in to 2 M NaOH (10 mL) and the aqueous layer extracted with Et_2_O (3 x 10 mL). The combined organic layers were dried (MgSO_4_) and the solvent evaporated under reduced pressure. The resulting white semi-solid was purified by chromatography (33% EtOAc / petrol), to give **23** as a clear oil (0.12 g, 90%); [α]_D_ –7.37 (*c* 0.38, CHCl_3_); ν_max_ (film) 3071, 3064, 3002, 2956, 2924, 2858, 1632, 1446, 1304, 1145, 1085 and 690 cm^–1^; δ_H_ (300 MHz) 7.92 (2H, d, *J =* 7.0 Hz, *ortho*-sulfone), 7.68 (1H, t, *J =* 7.5 Hz, *para*-sulfone), 7.59 (2H, t, *J =* 7.5 Hz, *meta*-sulfone), 5.71 (1H, dddd (ddt), *J =* 17.0, 10.0, 6.5, 6.5 Hz, H-4'), 4.96 (1H, dd, *J =* 10.0, 2.0 Hz, H-5'), 4.92 (1H, dd, *J =* 7.0, 2.0 Hz, H-5'), 3.61-3.54 (1H, m, H-2), 3.23 (1H, ddd, *J =* 10.0, 7.0, 3.0 Hz, H-5), 3.18-3.08 (1H, m, H-3), 2.37 (1H, ddd, *J =* 14.0, 6.0, 3.0 Hz, H-4), 1.98 (1H, m, H-4), 1.61-1.23 (12H, m, 6 x CH_2_), 0.89 (3H, m, Me); δ_C_ (75 MHz) 138.23, 133.71, 129.31, 128.42, 127.95, 126.43, 114.78, 68.83, 59.50, 58.69, 35.96, 35.18, 34.44, 33.47, 29.34, 26.13, 22.58, 13.95; *m/z* (CI) 671 [2M+H]^+^, 336 [M+H]^+^, 298, 266, 236, 194, 136 (Found: [M+H]^+^, 336.197891. C_19_H_29_NO_2_S requires [M+H]^+^, 336.199726).

**(2*R*,3*S*,5*R*)-1-benzoyl-5-(methylethyl)-2-(4'-pentenyl)-3-(phenylsulfonyl)pyrrolidine (14c).**

To a solution of (3*R*)-6-acetoxy-3-benzoylamino-2-methyl-5-(phenylsulfonyl)-10-undecene (2.20 g, 4.54 mmol, 1.0 eq.) in THF 137.5 mL) under a nitrogen atmosphere was added *t*-BuOH (4.28 mL, 45.36 mmol, 10.0 eq.) and *t*-BuOK (1M in THF; 9.30 mL, 9.30 mmol, 2.05 eq.). The cloudy dark red solution was stirred at rt overnight and then poured in to a saturated NH_4_Cl solution (50 mL). The two phases were separated and the aqueous phase extracted with EtOAc (3 x 50 mL). The combined organic layers were dried (MgSO_4_) and concentrated under reduced pressure. The resultant immobile orange oil was purified by chromatography (33% EtOAc / petrol) to give **14c** as a crystalline solid (1.35 g, 70%); mp 143-145˚C; [α]_D_ –28.86 (*c* 0.4, CH_2_Cl_2_); ν_max_ (CH_2_Cl_2_ film) 3062, 2961, 2933, 2871, 1641, 1582, 1580, 1456, 1446, 1403, 1386, 1357, 1306, 1147, 1085, 734, 703, 690 and 666 cm^–1^; δ_H_ (500 MHz) doubled due to rotamers 7.97-7.41 (20H, brm, Ar-H), 5.61 (1H, brm H-2'), 5.52 (1H, brm, H-2'), 4.88 (2H, d, *J =* 10.0 Hz, H-1'), 4.81 (2H, brm, H-1'), 4.59 (1H, brm, H-2), 4.48 (1H, brm, H-2), 4.20 (1H, brm, H-5), 3.92 (1H, brm, H-5), 3.36 (1H, brm, H-3), 3.27 (1H, brm, H-3), 2.64 (2H, dd, *J =* 14.5, 8.5 Hz, H-4), 2.12 (2H, brm, H-4), 1.87 (2H, brm, CH), 1.63 (2H, brm CH_2_), 1.33 (2H, brm, CH_2_), 1.09-0.61 (20H, brm, 2 x Me & 2 x CH_2_); δ_C_ (75 MHz) broad peaks 137.51, 137.13, 134.03, 129.40, 128.62, 128.34, 126.45, 114.96, 66.58, 65.99, 63.28, 62.29, 60.36, 59.47, 35.78, 33.86, 32.76, 30.13, 26.76, 25.12, 19.81, 19.39, 17.88, 15.45, 14.18; *m/z* (CI) 426 [M+H]^+^, 286, 242, 216, 180, 160, 122, 105 (Found: [M+H]^+^, 426.212137. C_25_H_31_NO_3_S requires [M+H]^+^, 426.210291); (Found C, 70.81; H, 6.99; N, 3.27. C_25_H_31_NO_3_S requires C, 70.59; H, 7.29; N, 3.29%).

**(2*R*,3*S*,5*R*)-5-(Methylethyl)-2-(4'-pentenyl)-3-(phenylsulfonyl)pyrrolidine (15c).**

To a solution of (2*R*,3*S*,5*R*)-1-benzoyl-5-(methylethyl)-2-(4'-pentenyl)-3-(phenylsulfonyl)pyrrolidine **14c** (0.14 g, 0.33 mmol, 1.0 eq.) in THF (3.28 mL) at –78 °C under a nitrogen atmosphere was added DIBAL (1.5 M in toluene; 0.44 mL, 0.66 mmol, 2.0 eq.). The solution was stirred at ––78 °C for 4 h and at rt for 2 h before MeOH was added to destroy unreacted DIBAL. The solution was poured into a vigorously stirred slurry of EtOAc and NaHCO_3_ and NaHCO_3_ added until a granular precipitate was formed. The slurry was filtered and the residue washed with EtOAc. The combined EtOAc layers were evaporated under reduced pressure to give a cloudy oil which was purified by chromatography (33% EtOAc / petrol) to give **15c** as a clear oil (0.07 g, 74%); [α]_D_ +22.12 (*c* 0.85, CH_2_Cl_2_); ν_max_ (neat) 3068, 2956, 2929, 2870, 1531, 1446, 1414, 1387, 1369, 1304, 1146, 1086, 910, 737 and 690 cm^–1^; δ_H_ (300 MHz) 7.92 (2H, d, *J =* 7.0 Hz, *ortho*-sulfone), 7.69 (1H, t, *J =* 7.5 Hz, *para*-sulfone), 7.60 (2H, t, *J =* 7.5 Hz, *meta*-sulfone), 5.72 (1H, dddd (ddt), *J =* 17.0, 10.0, 6.5, 6.5 Hz, H-4'), 5.00-4.91 (2H, m, 2 x H-5'), 3.58-3.53 (1H, m, H-2), 3.22 (1H, ddd, *J =* 10.5, 7.0, 3.0 Hz, H-5), 2.85 (1H, ddd, *J =* 10.0, 8.0, 6.0 Hz, H-3), 2.34 (1H, ddd, J 14.0, 6.0, 3.0 Hz, H-4), 1.99-1.97 (2H, m, H-4 & CH of *i*Pr), 1.64-1.27 (6H, m, 3 x CH_2_), 0.95 (3H, d, *J =* 6.5 Hz, Me), 0.89 (3H, d, *J =* 6.5 Hz, Me); δ_C_ (75 MHz) 138.75, 138.31, 133.74, 129.33, 128.61, 114.76, 68.72, 64.87, 59.27, 36.14, 33.66, 33.54, 32.28, 29.07, 20.41, 19.59; *m/z* (CI) 643 [2M+H]^+^, 322 [M+H]^+^, 286, 252, 180, 160, 136, 126, 80; (Found: [M+H]^+^, 322.182611. C_18_H_27_NO_2_S requires [M+H]^+^, 322.184076).

**(2*R*,3*S*,5*R*)-1-Benzyl-5-(methylethyl)-2-(4'-pentenyl)-3-(phenylsulfonyl)-pyrrolidine (16c).**

Compound **16b** was synthesized in an analogous fashion to the preparation of compound **28**; mp 61–62 °C; [α]_D_ +32.03 (*c* 3.55, CH_2_Cl_2_); ν_max_ (CH_2_Cl_2_ film) 3050, 3028, 2955, 2929, 2869, 1446, 1305, 1147, 1085, 1027, 997, 912, 732, 700 and 690 cm^–1^; δ_H_ (300 MHz) 7.90 (2H, d, *J =* 8.0 Hz, *ortho*-sulfone), 7.67 (1H, t, *J =* 7.5 Hz, *para*-sulfone), 7.58 (2H, t, *J =* 7.5 Hz, *meta*-sulfone), 7.43-7.22 (5H, m, Bn-Ar), 5.59 (1H, dddd (ddt), *J =* 13.5, 11.5, 6.5, 6.5 Hz, H-4'), 4.87-4.80 (2H, m, 2 x H-5'), 3.85 (1H, d, *J =* 14.0 Hz, H of Bn), 3.67 (1H, d, *J =* 14.0 Hz, H of Bn), 3.33-3.23 (2H, m, H-2 & H-5), 2.91 (1H, dd, *J =* 6.5, 4.5 Hz, H-3), 2.14 (1H, ddd, *J =* 14.0, 6.5, 3.0 Hz, H-4), 1.87-1.63 (7H, m, H-4 & 3 x CH_2_), 1.24-1.14 (1H, m, CH of *i*Pr), 0.86 (3H, d, *J =* 7.0 Hz, Me), 0.83 (3H, d, *J =* 7.0 Hz, Me); δ_C_ (75 MHz) 139.66, 138.43, 138.23, 133.56, 129.13, 128.98, 128.62, 127.99, 126.79, 126.79, 114.28, 68.70, 66.98, 64.54, 58.22, 34.81, 33.27, 28.90, 25.98, 23.52, 20.02, 15.24; *m/z* (CI) 412 [M+H]^+^, 368, 322, 272, 226, 202, 180, 160, 136, 106, 91, 52; (Found: [M+H]^+^, 412.235045. C_25_H_33_NO_2_S requires [M+H]^+^, 412.231026); (Found C, 72.89; H, 8.05; N, 3.22. C_25_H_33_NO_2_S requires C, 72.99; H, 8.03; N, 3.41%).

**(1*R*,3*R*,5*S*,9*R*)-3-Butyl-5-methyl-1-(phenylsulfonyl)-indolizidine (24-*syn*) and (1*R*,3*R*,5*R*,9*R*)-3-butyl-5-methyl-1-(phenylsulfonyl)indolizidine (24-*anti*).**

To a bright yellow suspension of Hg(OAc)_2_ (0.19 g, 0.60 mmol, 1.1 eq.) in 1:1 THF:H_2_O (1.2 mL) was added a solution of (2*S*,3*R*,5*R*)-5-butyl-2-(4'-pentenyl)-3-(phenylsulfonyl)pyrrolidine **23** (0.18 g, 0.55 mmol, 1.0 eq.) in THF (1.1 mL) dropwise. The suspension cleared to a pale yellow solution and was stirred at rt for 3 h, at which point a white suspension had formed to which was added NaBH_4_ (2.5 M in NaOH; 0.16 mL, 0.41 mmol, 0.75 eq.). A grey precipitate was formed. The solution was partitioned between H_2_O (10 mL) and Et_2_O (10 mL). The aqueous layer was extracted with Et_2_O (3 x 10 mL) and the combined organic layers dried (MgSO_4_) and concentrated under reduced pressure. The crude semi-solid was purified by chromatography (33% EtOAc / petrol), to give a 1.0:2.5 mixture of (1*R*,3*R*,5*S*,9*R*)-3-butyl-5-methyl-1-(phenylsulfonyl)indolizidine **24-*syn*** as a crystalline solid (0.05 g, 28%) and an inseparable mixture of (1*R*,3*R*,5*R*,9*R*)-3-butyl-5-methyl-1-(phenylsulfonyl)indolizidine **24*-anti*** and (2*S*,3*R*,5*R*)-5-butyl-2-(4'-pentenyl)-3-(phenylsulfonyl)pyrrolidine **23** as a clear oil (0.12 g, 68%); (1*R*,3*R*,5*S*,9*R*)-3-butyl-5-methyl-1-(phenylsulfonyl)indolizidine **24-*syn***; mp 61 °C; [α]_D_^32^ –29.67 (*c* 1.38, CHCl_3_); ν_max_ (CH_2_Cl_2_ film) 2955, 2930, 2859, 1446, 1303, 1145, 1086 and 582 cm^–1^; δ_H_ (500 MHz) 7.89 (2H, d, *J =* 8.0 Hz, *ortho*-sulfone), 7.67 (1H, t, *J =* 7.5 Hz, *para*-sulfone), 7.58 (2H, t, *J =* 7.5 Hz, *meta*-sulfone), 3.31 (1H, q, *J =* 10.0 Hz, H-1), 2.65-2.61 (1H, m, H-3), 2.56 (1H, td, *J =* 10.0, 4.0 Hz, H-9), 2.32-2.24 (2H, m, H-2 & H-5), 1.95 (1H, dd, *J =* 10.0, 2.0 Hz, H-8), 1.71-1.65 (2H, m, H-7 & H-8), 1.57-1.48 (2H, m, H-2 & H-7), 1.31-1.13 (8H, m, 4 x CH_2_), 1.11 (3H, d, *J =* 6.0 Hz, Me-5), 0.85 (3H, t, *J =* 7.0 Hz, Me); δ_C_ (75 MHz) 139.06, 133.73, 129.25, 128.41, 65.88, 65.51, 61.13, 38.75, 35.37, 32.29, 30.52, 28.58, 24.24, 22.78, 14.07; *m/z* (CI) 336 [M+H]^+^, 316, 278, 194, 152, 136, 122, 94, 80 (Found: [M+H]^+^, 336.199502. C_19_H_29_NO_2_S requires [M+H]^+^, 336.199726).

**Isolation of (1*R*,3*R*,5*R*,9*R*)-3-butyl-5-methyl-1-(phenylsulfonyl)indolizidine (24-*anti*).**

To a solution of (1*R*,3*R*,5*R*,9*R*)-3-butyl-5-methyl-1-(phenylsulfonyl)indolizidine **24-*anti*** and (2*S*,3*R*,5*R*)-5-butyl-2-(4'-pentenyl)-3-(phenylsulfonyl)pyrrolidine **23** (0.11 g, 0.33 mmol, 1.0 eq.) in CH_2_Cl_2_ (3.3 mL) at 0 °C under a nitrogen atmosphere was added pyridine (0.02 mL, 0.26 mmol, 0.8 eq.) and benzoyl chloride (0.03 mL, 0.26 mmol, 0.8 eq.). The solution was stirred at rt overnight whereupon it was cooled to 0 °C and DMPA (0.02 mL, 0.20 mmol, 0.6 eq.) was added. The solution was stirred at rt for 30 min and partitioned between H_2_O (10 mL) and Et_2_O (10 mL). The aqueous layer was extracted with Et_2_O (3 x 10 mL) and the combined organic layers washed repeatedly with saturated NH_4_Cl solution (3 x 10 mL), brine (3 x 10 mL) and dried (MgSO_4_). The solvent was evaporated under reduced pressure to give a semi-solid which was purified by chromatography (33% EtOAc / petrol) to give 1:10 mixture of (2*S*,3*R*,5*R*)-1-benzoyl-5-butyl-2-(4'-pentenyl)-3-(phenylsulfonyl)pyrrolidine **22** (0.01 g, 8%) and (1*R*,3*R*,5*R*,9*R*)-3-butyl-5-methyl-1-(phenylsulfonyl)indolizidine **24-*anti*** (0.08 g, 80%) as a coloured immobile oil; [α]_D_^32^ –58.24 (*c* 0.63, CHCl_3_); ν_max_ (CH_2_Cl_2_ film) 3064, 2955, 2931, 2859, 2831, 1446, 1379, 1304, 1270, 1314, 1227, 1193, 1147, 1087, 1076, 761, 746, 715 and 690 cm^–1^; δ_H_ (500 MHz) 7.89 (2H, d, *J =* 7.5 Hz, *ortho*-Ar), 7.65 (1H, t, *J =* 7.5 Hz, *para*-sulfone), 7.57 (2H, t, *J =* 7.5 Hz, *meta*-sulfone), 3.33 (1H, brt, *J =* 6.0 Hz, H-5), 3.15 (1H, ddd, *J =* 10.5, 8.5, 4.0 Hz, H-1), 2.96 (1H, td, *J =* 11.0, 2.5 Hz, H-9), 2.59 (1H, ddd, *J =* 16.5, 9.5, 3.0 Hz, H-3), 2.24 (1H, ddd, *J =* 13.5, 7.0, 4.5 Hz, H-2), 1.66-1.51 (3H, m, H-2, H-8 & H of CH_2_), 1.48-1.39 (2H, m, CH_2_), 1.38-1.09 (8H, m, 4 x CH_2_), 0.90 (3H, d, *J =* 6.5 Hz, Me-5), 0.87 (3H, t, *J =* 7.0 Hz, Me); δ_C_ (75 MHz) 139.20, 133.69, 129.27, 128.48, 66.38, 58.06, 66.38, 58.06, 47.58, 32.06, 31.39, 30.82, 28.04, 23.02, 19.03, 14.03, 7.70; *m/z* (CI) 336 [M+H]^+^, 320, 278, 192, 160, 136, 126, 110, 94, 80 (Found: [M+H]^+^, 336.198446. C_19_H_29_NO_2_S requires [M+H]^+^, 336.199726).

**(1*S*,3*R*,5*S*,9*R*)-5-Methyl-3-(methylethyl)-1-(phenylsulfonyl)indolizidine (26-*anti*) and (1*S*,3*R*,5*R*,9*R*)-5-methyl-3-(methylethyl)-1-(phenylsulfonyl)indolizidine (26-*syn*).**

To a bright yellow solution of Hg(OAc)_2_ (0.060 g, 0.19 mmol, 1.05 eq.) in 1:1 H_2_O:THF (0.40 mL) was added (2*R*,3*S*,5*R*)-5-(methylethyl)-2-(4'-pentenyl)-3-(phenylsulfonyl)-pyrrolidine **15c** (0.058 g, 0.18 mmol, 1.0 eq.) in THF (0.36 mL) dropwise. The considerably paler suspension was stirred at rt for 40 min until the suspension was virtually white. NaBH_4_ (2.5 M in NaOH; 0.053 mL, 0.13 mmol, 0.75 eq.) was added causing a grey precipitate to form. The suspension was partitioned between H_2_O (10 mL) and EtOAc (10 mL). The aqueous layer was extracted with EtOAc (3 x 10 mL) and the combined organics dried (MgSO_4_). The solvent was under reduced pressure to give after chromatography (33% EtOAc-petrol) a 5:1 mixture of **26-*anti*** and **26-*syn*** (0.022 g, 38%) separable by HPLC only, and starting material (0.023 g, 40%); minor compound (1*S*,3*R*,5*R*,9*R*)-5-methyl-3-(methylethyl)-1-(phenylsulfonyl)indolizidine **26-*syn***; mp 94-95 °C; [α]_D_ +11.96 (*c* 0.92, CHCl_3_); ν_max_ (film) 2962, 2931, 2867, 1446, 1321, 1147, 1085, 720 and 608 cm^–1^; δ_H_ (300 MHz) 7.88 (2H, d, *J =* 7.0 Hz, *ortho*-sulfone), 7.66 (1H, t, *J =* 7.5 Hz, *para*-sulfone), 7.57 (2H, t, *J =* 7.5 Hz, *meta*-sulfone), 3.16 (1H, td, *J =* 10.5, 8.0 Hz, H-1), 2.74 (1H, dt, *J =* 10.5, 3.0 Hz, H-3), 2.58 (1H, td, *J =* 10.5, 2.0 Hz, H-9), 2.36-2.30 (1H, m, H-5), 2.00-1.91 (2H, m, H-2 & H-8), 1.85-1.81 (1H, m, CH of *i*Pr), 1.68-1.63 (1H, m, H-2), 1.51-1.44 (1H, m, H-8), 1.32-1.10 (4H, m, 2 x H-6 & 2 x H-7), 1.04 (3H, d, *J =* 6.5 Hz, Me-5), 0.72 (3H, d, *J =* 7.0 Hz, Me of *i*Pr), 0.71 (3H, d, *J =* 7.0 Hz, Me of *i*Pr); δ_C_ (75 MHz) 133.70, 130.51, 129.22, 128.40, 65.91, 60.05, 35.45, 31.98, 29.70, 26.82, 25.65, 24.28, 22.27, 19.92, 14.72; *m/z* (CI) 322 [M+H]^+^, 278, 180, 136; (Found: [M+H]^+^, 322.185979. C_18_H_27_NO_2_S requires [M+H]^+^, 322.184076); major compound (1*S*,3*R*,5*S*,9*R*)-5-methyl-3-(methylethyl)-1-(phenylsulfonyl)indolizidine **26-*anti***; mp 105-106 °C; [α]_D_ +46.77 (*c* 0.33, CHCl_3_); ν_max_ (film) 2967, 2950, 2929, 2859, 2848, 2807, 1463, 1450, 1375, 1358, 1328, 1305, 1284, 1246, 1242, 1202, 1181, 1145, 1124, 1086, 1070, 997, 896, 767, 745, 719 and 694 cm^–1^; δ_H_ (300 MHz) 7.90 (2H, dd, *J =* 7.0, 2.0 Hz, *ortho*-sulfone), 7.65 (1H, t, *J =* 7.5 Hz, *para*-sulfone), 7.57 (2H, t, *J =* 7.5 Hz, *meta*-sulfone), 3.19 (1H, m, H-5), 3.09-3.04 (1H, m, H-1), 2.98 (1H, td, *J =* 10.0, 2.5 Hz, H-9), 2.64 (1H, td, *J =* 13.0, 4.0 Hz, H-3), 1.93 (1H, ddd, *J =* 13.0, 7.5, 4.0 Hz, H-2), 1.85 (1H, quintetd, *J =* 7.0, 4.0 Hz, CH of *i*Pr), 1.64-1.54 (3H, m, H-2, H-6 & H-7), 1.48-1.38 (3H, m, H-6, H-7 & H-8), 1.11 (1H, ddd, *J =* 15.0, 11.0, 5.0 Hz, H-8), 0.87 (3H, d, *J =* 6.5 Hz, Me-5), 0.83 (3H, d, *J =* 7.0 Hz, Me of *i*Pr), 0.70 (3H, d, *J =* 7.0 Hz, Me of *i*Pr); δ_C_ (75 MHz) 135.99, 131.58, 130.83, 68.86, 64.25, 58.29, 49.42, 34.58, 33.27, 28.23, 27.23, 22.17, 21.45, 16.36, 9.90; *m/z* (CI) 322 [M+H]^+^, 278, 180, 136, 94 (Found: [M+H]^+^, 322.183777. C_18_H_27_NO_2_S requires [M+H]^+^, 322.184076).

**(2*S*,3*R*,5*R*)-1-benzyl-5-butyl-2-(4'-pentenyl)-3-(phenylsulfonyl)pyrrolidine (28).**

To a solution of (2*S*,3*R*,5*R*)-1-benzoyl-5-butyl-2-(4'-pentenyl)-3-(phenylsulfonyl)-pyrrolidine **22** (1.31 g, 2.99 mmol, 1.0 eq.) in CH_2_Cl_2_ (30 mL) at 0 °C under a nitrogen atmosphere was added DIBAL (1.5 M in toluene; 7.96 mL, 11.93 mmol, 4.0 eq.). The solution was stirred at 0 °C for 30 min. and at rt for 2 h, whereupon MeOH was carefully added until no more effervescence was observed. The solution was poured in to a vigorously stirred slurry of NaHCO_3_ and EtOAc and NaHCO_3_ added until a granular precipitate was observed. The slurry was filtered and washed with EtOAc and the solvent evaporated under reduced pressure. The crude oil was purified by chromatography (33% EtOAc / petrol) to give **28** as a clear oil (1.19 g, 94%); [α]_D_^32^ –30.50 (*c* 2.10, CHCl_3_); ν_max_ (film) 3058, 3028, 3002, 3084, 2928, 2858, 1494, 1446, 1305, 1147, 1085, 910, 730, 699 and 690 cm^–1^; δ_H_ (300 MHz) 7.89 (2H, d, *J =* 7.0 Hz, *ortho*-sulfone), 7.67 (1H, t, *J =* 7.5 Hz, *para*-sulfone), 7.57 (2H, t, *J =* 7.5 Hz, *meta*-sulfone), 7.31-7.22 (5H, m, Bn Ar-H), 5.59 (1H, dddd (ddt), *J =* 16.5, 9.5, 7.0, 7.0 Hz, H4'), 4.86 (2H, d, *J =* 12.0 Hz, 2 x H-5'), 3.85 (1H, d, *J =* 14.0 Hz, H of Bn), 3.67 (1H, d, *J =* 14.0 Hz, H of Bn), 3.30-3.28 (2H, m, H-2 & H-5), 2.89-2.87 (1H, m, H-3), 2.36 (1H, dd, *J =* 12.5, 6.0 Hz, H-4), 1.82-1.60 (4H, m, H-4 & 3 x H of CH_2_), 1.27-1.02 (9H, m, 9 x H of CH_2_), 0.86 (3H, t, *J =* 6.5 Hz, Me); δ_C_ (75 MHz) 139.91, 138.55, 133.67, 129.24, 128.87, 128.81, 128.12, 126.88, 114.45, 67.10, 64.82, 63.96, 57.70, 34.69, 34.59, 33.51, 32.22, 28.11, 23.60, 22.91, 14.01; *m/z* (CI) 426 [M+H]^+^, 368, 356, 336, 300, 286, 194, 160, 138, 126, 91 (Found: [M+H]^+^, 426.247383. C_26_H_35_NO_2_S requires [M+H]^+^, 426.246677); (Found C, 73.18; H, 8.45; N, 3.26. C_26_H_35_NO_2_S requires C, 73.41; H, 8.24; N, 3.29%).

**(1*S*,3*R*,5*R*,9*R*)-5-Methyl-3-(methylethyl)-1-(phenylsulfonyl)indolizidine (26-*syn*).**

To a flask charged with PdCl_2_ (0.009 g, 0.05 mmol, 0.1 eq.) and CuCl (0.052 g, 0.52 mmol, 1.0 eq.) under an oxygen atmosphere was added 7 : 1 DMF : H_2_O (0.9 mL). The resultant dark yellow solution was stirred at rt for 1 h and gradually became a dark green colour. A solution of (2*R*,3*S*,5*S*)-1-benzyl-5-(methylethyl)-2-(4'-pentenyl)-3-(phenylsulfonyl)pyrrolidine **16c** (0.214 g, 0.52 mmol, 1.0 eq.) in 7 : 1 DMF : H_2_O (1.0 mL) was added to give a light green solution which was stirred at rt for 24 h. The black solution was quenched with NaHCO_3_ and filtered. The filtrate was partitioned between H_2_O (10 mL) and EtOAc (10 mL). The aqueous layer was extracted with EtOAc (3 x 10 mL) and the combined organics dried (MgSO_4_) and concentrated under reduced pressure. The red oil was purified by preparative tlc (33% EtOAc / petrol) to give (2*R*,3*S*,5*R*)-1-benzyl-5-(methylethyl)-2-(4'-oxo-pentyl)-3-(phenylsulfonyl)pyrrolidine as a clear oil (0.13 g, 61%); ν_max_ (neat) 3085, 3062, 3028, 3000, 2955, 2931, 2872, 1714, 1494, 1447, 1388, 1361, 1305, 1147, 1085, 754, 734, 701 and 692 cm^–1^; δ_H_ (300 MHz) 7.89 (2H, d, *J =* 7.0 Hz, *ortho*-sulfone), 7.65 (1H, t, *J =* 7.5 Hz, *para*-sulfone), 7.57 (2H, t, *J =* 7.5 Hz, *meta*-sulfone), 7.30-7.23 (5H, m, Ar-H), 3.86 (1H, d, *J =* 13.5 Hz, H of Bn), 3.63 (1H, d, *J =* 13.5 Hz, H of Bn), 3.33-3.27 (2H, m, H-2 & H-5), 2.94-2.88 (1H, m, H-3), 2.13-2.02 (2H, m, H-4 & H-1'), 1.99 (3H, s, Me'), 1.85-1.77 (2H, m, H-4 & H-1'), 1.39-1.10 (5H, m, CH, 2 x H-2' & 2 x H-3'), 0.86 (3H, d, *J =* 6.5 Hz, Me), 0.83 (3H, d, *J =* 6.5 Hz, Me); δ_C_ (75 MHz) 208.59, 139.80, 138.37, 133.70, 129.29, 129.20, 128.70, 128.14, 126.98, 68.97, 66.91, 64.29, 58.40, 43.03, 34.90, 29.82, 29.08, 26.24, 20.13, 18.50, 15.45; *m/z* (CI) 428 [M+H]^+^, 412, 384, 338, 288, 244, 218, 202, 196, 160, 106, 52 (Found: [M+H]^+^, 428.226763. C_25_H_32_NO_3_S requires [M+H]^+^, 428.225941).

To a suspension of (2*R*,3*S*,5*R*)-1-benzyl-5-(methylethyl)-2-(4'-oxo-pentyl)-3-(phenylsulfonyl)pyrrolidine (0.15 g, 0.34 mmol, 1.0 eq.) and 10% Pd / C (0.15 g) in MeOH (3.40 mL) under a nitrogen atmosphere was added 1,4-cyclohexadiene (0.48 mL, 5.10 mmol, 15.0 eq.). The suspension was heated to reflux for 6 h at which point tlc revealed reaction had gone to completion. The solution was cooled to rt and filtered through celite and the residue washed with EtOAc. The organic filtrate was evaporated under reduced pressure to give a red oil. Purification by chromatography (33% EtOAc / petrol) gave (1*S*,3*R*,5*R*,9*R*)-5-methyl-3-(methylethyl)-1-(phenylsulfonyl)indolizidine **26-*syn*** as an oily solid (0.028 g, 30%); identical in every way to material prepared previously.

**(2*S*,3*R*,5*R*)-1-Benzyl-5-butyl-2-(4'-oxo-pentyl)-3-(phenylsulfonyl)pyrrolidine (29).**

To a solution of (2*S*,3*R*,5*R*)-1-benzyl-5-butyl-2-(4'-pentenyl)-3-(phenylsulfonyl)-pyrrolidine **28** (1.18 g, 2.78 mmol, 1.0 eq.) in 3 : 1 THF : H_2_O (14.0 mL) was added Hg(OAc)_2_ (0.93 g, 2.92 mmol, 1.05 eq.) to give a bright yellow solution which was stirred at rt for 2 h. The solution was then added to a solution of PdCl_2_ (0.32 g, 1.81 mmol, 0.65 eq.) and CuCl_2_ (1.14 g, 8.47 mmol, 3.05 eq.) in THF (9.0 mL). The brown solution became a dark green solution and black precipitate appeared almost instantly. The solution was stirred at rt for 1.5 h after which saturated NaHCO_3_ solution (10 mL) was added, resulting in effervescence. The layers were separated and the aqueous phase diluted with H_2_O and 2 M NaOH before being extracted with Et_2_O (4 x 20 mL). The combined organics were dried (MgSO_4_) and concentrated under reduced pressure. The yellow semi-solid was purified by chromatography (33% EtOAc / petrol) to give (2*S*,3*R*,5*R*)-1-benzyl-5-butyl-2-(4'-oxo-pentyl)-3-(phenylsulfonyl)pyrrolidine **29** as a clear oil (0.86 g, 70%); [α]_D_ –29.74 (*c* 0.94, CHCl_3_); ν_max_ (film) 3058, 3028, 2954, 2930, 2876, 1714, 1447, 1361, 1305, 1147, 1085, 753, 736, 700 and 692 cm^–1^; δ_H_ (300 MHz) 7.89 (2H, dd, *J =* 7.0, 1.5 Hz, *ortho*-sulfone), 7.66 (1H, t, *J =* 7.5 Hz, *para*-sulfone), 7.57 (2H, t, *J =* 7.0 Hz, *meta*-sulfone), 7.31-7.23 (5H, m, Ar-H), 3.88 (1H, d, *J =* 14.0 Hz, H of Bn), 3.63 (1H, d, *J =* 14.0 Hz, H of Bn), 3.38-3.32 (1H, m, H-2), 3.29 (1H, q, *J =* 6.5 Hz, H-5), 2.83-2.91 (1H, m, H-3), 2.29 (1H, ddd, *J =* 14.0, 6.0, 2.5 Hz, H-4), 2.18-1.97 (3H, m, H-4 & 2 x H-3'), 2.01 (3H, s, MeCO), 1.75-1.57 (3H, m, 3 x H of CH_2_), 1.45-1.41 (1H, m, H of CH_2_), 1.25-1.14 (6H, m, 3 x CH_2_), 0.86 (3H, t, *J =* 7.0 Hz, Me); δ_C_ (68 MHz) 208.55, 139.89, 138.36, 133.62, 129.21, 128.86, 128.68, 128.07, 126.86, 66.64, 64.46, 64.00, 57.63, 43.10, 34.47, 34.36, 32.19, 29.78, 28.03, 22.84, 18.24, 13.98; *m/z* (CI) 442 [M+H]^+^, 356, 338, 336, 278, 136, 110, 91, 68 (Found: [M+H]^+^, 442.245645. C_26_H_35_NO_3_S requires [M+H]^+^, 442.241591).

**(1*R*,3*R*,5*S*,9*R*)-3-Butyl-5-methyl-1-(phenylsulfonyl)indolizidine (24-*syn*).**

To a suspension of (2*S*,3*R*,5*R*)-1-benzyl-5-butyl-2-(4'-oxo-pentyl)-3-(phenylsulfonyl)-pyrrolidine **29** (0.05 g, 0.12 mmol, 1.0 eq.) and 10% Pd / C (0.05 g) in MeOH (1.2 mL) under a nitrogen atmosphere was added 1,4-cyclohexadiene (0.18 mL, 1.87 mmol, 15.0 eq.). The solution was heated to reflux for 5 h. The reaction was cooled to r.t. and filtered through celite and the residue washed with EtOAc. The combined filtrate and washings were evaporated under reduced pressure to give, with out further purification, (1*R*,3*R*,5*S*,9*R*)-3-butyl-5-methyl-1-(phenylsulfonyl)indolizidine **24-*syn*** as a slowly solidifying oil (0.036 g, 89%); identical in every way to material prepared previously.

**(3*R*,5*S*,9*R*)-3-butyl-5-methylindolizidine, (+)-monomorine I.**

To a solution of (1*R*,3*R*,5*S*,9*R*)-3-butyl-5-methyl-1-(phenylsulfonyl)indolizidine **24-*syn*** (0.035 g, 0.10 mmol, 1.0 eq.) in THF (2.1 mL) at rt under a nitrogen atmosphere was added sodium naphthalenide (*c*. 1 M in THF; 0.35 mL, 0.35 mmol, 3.5 eq.) dropwise. The solution rapidly became dark red. After 5 min NH_4_Cl was added with vigorous stirring. The colour faded to a pale yellow. The reaction was poured in to 1 M HCl (10 mL) and washed with Et_2_O (5 mL). The aqueous phase was basified to pH 11 with 2 M NaOH and the basic aqueous layer extracted with Et_2_O (3 x 5 mL). The combined organic layers were dried (MgSO_4_) and the solvent removed by evaporation under a stream of nitrogen. The yellow residue was purified by chromatography (alumina) (17% ether / hexane) to give (3*R*,5*S*,9*R*)-3-butyl-5-methylindolizidine, (+)-monomorine I, as a clear oil (0.011 g, 55%); [α]_D_^30^ +35.5 (*c* 0.66, hexane) (lit. +35.7);[3] ν_max_ (CH_2_Cl_2_) 2951, 2929, 2873, 2859, 2815, 2798, 2790, 2776, 1463, 1455, 1442, 1378, 1318, 1301, 1207 and 1124 cm^–1^; δ_H_ (300 MHz) 2.48 (1H, m, H-3), 2.24-2.21 (1H, m, H-5), 2.09-2.04 (1H, m, H-9), 1.89-1.62 (5H, m, H-1, H-2, H-7, H-8 & H-1'), 1.56-1.36 (3H, m, H-1, H-2 & H-6), 1.33-1.17 (8H, m, H-6, H-7, H-8, H-1', 2 x H-2' & 2 x H-3'), 1.15 (3H, d, *J =* 6.5 Hz, Me-5), 0.90 (3H, t, *J =* 7.0 Hz, Me-4'); δ_C_ (75 MHz) 67.19, 62.91, 60.27, 39.73, 35.90, 30.96, 30.36, 29.78, 29.38, 24.94, 22.92, 22.90, 14.15; *m/z* (CI) 196 [M+H]^+^, 194, 180, 138 (Found: [M+H]^+^, 196.206050. C_13_H_25_N requires [M+H]^+^, 196.206525). All data in agreement with the literature values.[3]

**References**

1. Hoye TR, Hanson PR, Vyvyan JR: *J Org Chem* 1994, **59:**4096-4103.

2. Perrin DD, Armarego WLF: *Purification of Laboratory Chemicals*: Pergamon Press; 1988.

3. Munchhof MJ, Meyers AI: *J Am Chem Soc* 1995, **117:**5399-5400.
